# Reply to Comment on “Reversible 3D optical data storage and information encryption in photo-modulated transparent glass medium”

**DOI:** 10.1038/s41377-022-00921-6

**Published:** 2022-07-26

**Authors:** Zhen Hu, Xiongjian Huang, Zhengwen Yang, Jianbei Qiu, Zhiguo Song, Junying Zhang, Guoping Dong

**Affiliations:** 1 College of Materials Science and Engineering, Kunming University of Science and Technology, 650093 Kunming, China; 2 State Key Laboratory of Luminescent Materials and Devices, School of Materials Science and Engineering, South China University of Technology, 510640 Guangzhou, China; 3 School of Physics, Beihang University, 100191 Beijing, China

**Keywords:** Optical data storage, Lithography

Dear Editor,

Tungsten-based photochromic materials are well known, such as tungsten–phosphate glasses, tungsten–tellurite glasses, and tungsten–borate glasses^1^. Photoluminescence glasses exhibit a wide range of application in the fields of display, lighting, laser and optical thermometry, et al. Combination of photochromic and luminescence can extend the application of luminescence materials^2–7^. Our focus is not on the development of new photochromic materials, but on the control of luminescence through photochromic reaction, especially achieved the real complex three-dimensional patterns using laser directly writing technology in photo-modulated transparent glass. In our work^2^, the three-dimensional optical data storage and information encryption application of photochromic glass with luminescence was obtained.

**Fig. 1:**
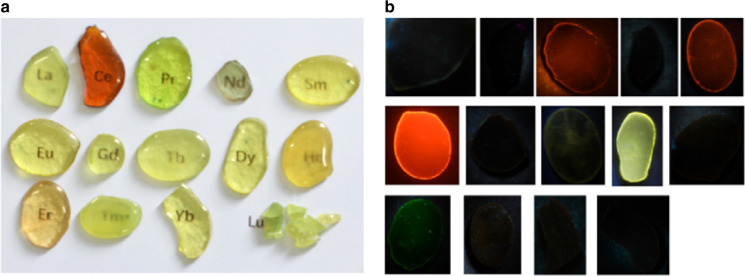
Photoluminescence properties of rare earth ions doped glass. **a** Photos of rare earth ions doped tungsten–phosphate glass with the molar compositions of 50WO_3_-39.5NaH_2_PO_4_-8BaF_2_-0.5Na_2_CO_3_-1Sb_2_O_3_-1(La, Ce, Pr, Nd, Sm, Eu, Gd, Tb, Dy, Ho, Er, Tm, Yb, Lu). **b** the luminescence photos of glasses with the molar compositions of 50WO_3_-39.5NaH_2_PO_4_-8BaF_2_-0.5Na_2_CO_3_-1Sb_2_O_3_-1(La, Ce, Pr, Nd, Sm, Eu, Gd, Tb, Dy, Ho, Er, Tm, Yb, Lu) under the excitation of 365 nm

Thank Poirier et al. for comment about importance of rare earth ions doped transparent photo-modulated glass. In our paper^2^, Poirier et al.’s work has been cited many times in the field of three-dimensional optical storage and photochromic mechanisms. Although our glass with the molar composition of 50WO_3_-39.5NaH_2_PO_4_-8BaF_2_-0.5Na_2_CO_3_-1Sb_2_O_3_-1EuF_3_ is similar to photochromic glass of composition (50WO_3_-40NaPO_3_-8.5BaF_2_-0.5Na_2_O-1Sb_2_O_3_)^8^, few rare earth ions doping have a significant influence on glass formation. As shown in Fig. 1a, the rare earth ions (La, Nd, Gd, Lu) doped glass is unstable and fragile, the rare earth ions (Ce, Sm, Tb, Ho, Tm) doped glass have poor transparency. The rare earth ions (Eu, Dy) doped glass have strong photoluminescence properties (Fig. 1b). As shown in Fig. 1, rare earth ions (Eu, Dy) doped glass have good transparency, stability and strong photoluminescence performance. The above results confirm that rare earth ions doping has a significant effect on the formation and photoluminescence properties of glasses.

In addition, Poirier et al. predicted the potential applications of optical data storage based on photochromic properties^8–11^. However, in the experiments they showed, only changed the color of the surface and the whole of photochromic glass by suing UV–Visible, and cannot write layered optical data and more complex three-dimensional holographic patterns in inside the glass^8,9^. Inspired by the above works, in our work^2^, we use 473 nm laser direct writing technique to write 3D optical information into the glass, illustrates the complex information model can be written in the modulation of light glass, read and erase, such as holographic logo design, QR code, binary data and complex three-dimensional structure. And the optical information can be stratified identification, so as to obtain encryption function. It shows its potential application in the field of information security. As they commented on our work, our novelty is that the luminescence of tungsten–phosphate photochromic glass doped with rare earth ions (Eu^3+^, Dy^3+^) is adjustable, which add to the way information can be read.

In summary, thanks for Poirier et al. extensive research in the field of photochromic glass. We apologize for not citing references about the composition and preparation method of glass.
